# Conformal and Disposable Antenna-Based Sensor for Non-Invasive Sweat Monitoring

**DOI:** 10.3390/s18124088

**Published:** 2018-11-22

**Authors:** Angie R. Eldamak, Elise C. Fear

**Affiliations:** 1 Department of Electronics and Electrical Communication Engineering, Faculty of Engineering, Ain Shams University, Cairo 11517, Egypt; 2 Schulich School of Engineering, University of Calgary, Calgary, AB T2N 1N4, Canada; fear@ucalgary.ca

**Keywords:** patch antenna, paper substrate, hydration sensor, sweat monitoring, disposable sensor, microwave sensing, biosensing

## Abstract

This paper presents a feasibility study for a non-wearable, conformal, low cost, and disposable antenna-based sensor for non-invasive hydration monitoring using sweat. It is composed of a patch antenna implemented on a cellulose filter paper substrate and operating in the range 2–4 GHz. The paper substrate can absorb liquids, such as sweat on the skin, through two slots incorporated within the antenna structure. Thus, the substrate dielectric properties are altered according to the properties of the absorbed liquid. Changes in reflection-based measurements are used to analyze salt solutions and artificial sweat, specifically the amount of sampled solution and the sodium chloride (NaCl) concentration. Using the shift in resonant frequency and magnitude of the reflection coefficient, NaCl concentrations in the range of 8.5–200 mmol/L, representing different hydration states, are detected. The measurements demonstrate the feasibility of using microwave based measurements for hydration monitoring using sweat.

## 1. Introduction

Health monitoring technologies have drawn significant attention from consumers and the scientific community in recent years [1,2,3]. These include heart rate, calorie count and daily exercise tracking, currently available in wearable fitness devices. Recently, hydration is emerging as a health indicator that requires non-invasive monitoring [3]. Hydration monitoring benefits athletes [4], active people, workers in hot environments, military personnel and older adults [5]. Hydration monitoring has the potential to improve personal health and contribute to health care cost reduction.

Dehydration can be defined as 1% or greater loss of body mass due to water loss [6,7]. Dehydration can result in impaired cognitive function, reduced physical performance, headaches and fatigue symptoms [6]. If dehydration becomes severe (loss of 8% of body weight), it may become fatal as reported in [8]. Dehydration can be classified in three major categories: hypertonic, hypotonic and isotonic, based on changes in water and sodium levels [9]. Hypertonic describes states where tissue loses more water than sodium, resulting in higher salt concentration [9,10]. This can be caused by inadequate fluid intake, sweating and vomiting [7]. If sodium is lost at a higher rate than water with the use of medications [6,7] or a genetic disorder [11], the body may experience hypotonic dehydration [12]. If the body lacks both water and sodium, isotonic dehydration occurs [7,9]. This loss is caused through perspiration, urine or diarrhea [7]. Dehydration can be monitored through weight changes [7], blood pressure [7], skin (stratum corneum) [9,13], saliva [14], urine and blood tests, as well as analysis of sweat [15]. 

Dehydrated persons produce sufficient sweat to be assessed. Sweat of dehydrated people, including athletes, was monitored after extensive exercise or exposure to excess heat in [15,16,17,18,19,20,21,22]. Quantitatively, 15–25% lower sweat rates are observed in a group of older subjects (age 52–71 years) compared to a younger group (age 20–30 years) subjected to similar environmental conditions and exercise [23,24,25,26,27]. Specifically, the average sweat rate for the younger group is reported as 0.4–1.2 μL/min/cm^2^ compared to 0.3–0.58 μL/min/cm^2^ for the older subjects with and without fluid replacement during exercise or subjected to heat. An average rate of 1 μL/min/cm^2^ of local sweat on the forearm would result in 0.4 L of estimated sweat loss after 20 min of exercise for an adult with weight of 70 kg, height 1.7 m and age 25 years [28,29]. For older age groups (above 80 years), measurement of axillary sweating or moisture is recommended in [30,31] to assess dehydration. However the exact amount of absorbed axillary sweating was not reported for older patients. 

Human sweat carries rich physiological biomarkers that make it an attractive fluid for non-invasive hydration monitoring. It includes electrolytes (sodium, chloride, potassium, magnesium, and calcium), metabolites, proteins and amino acids [15,16,17,18,19,32]. Beside traditional laboratory practices [7,15], several new technologies are reported in [16,17,19,20,21,22,33] to analyze sweat biomarkers for non-invasive monitoring. This includes non-selective technologies to monitor overall sweat properties, such as measuring sweat pH levels using bio-textiles sensors [16,17] or conductivity [19]. Other selective technologies record potential difference or impedance across sodium (Na^+^) selective electrodes (ISE) [20,33], or multi-biomarker sensing selective patches [21,22]. Most of the reported technologies require either a conditioning period, large sample size, additional electronic circuits or special sweat sampling mechanisms. A common theme in the different sensing works reported in [9,15,17,19,20,21,22,33] is that, among different sweat electrolytes, sodium (Na^+^) or sodium chloride (NaCl) results in the largest recorded variations with changing hydration states.

Recently, several studies reported assessment of human dehydration using microwave signals [9,34,35,36]. Body fluids with different electrolytic concentrations can result in different losses and hence absorption of electromagnetic waves [9]. The detected reflected or transmitted signals carry information on electrolytic concentrations, motivating development of microwave-based sensors for non-invasive hydration monitoring. Most of the reported studies used reflection or transmission measurements to assess water content in blood plasma [34,35,36]. On the other hand, none of these studies [34,35,36] analyzed sweat content or used microwaves to track electrolytes concentrations directly. However, Brendtke et al. developed a patch antenna in the range of 7.0–9.5 GHz for monitoring hydration status through assessing water content in skin tissues [9]. This work focuses on developing artificial skin equivalents incubated in a range of test liquids. Among these tested liquids, NaCl solutions with concentrations from 0% to 20% were used to alter skin hydration levels. The changes in Return Loss (RL) and frequency of the local minimum (ƒ_min_) were recorded for different skin equivalents, demonstrating RL magnitude changes of 3.4 dB and ƒ_min_ changes of 90 MHz with changing NaCl concentrations from 0% to 20%. This sensitivity is recorded when placing samples in a bioreactor chamber incorporating the antenna, and may be altered by changing measurement procedures, such as integration into a wearable module or when it is placed in direct contact on real skin. Moreover, the work in [9] does not include testing with other major electrolytes in body fluids.

This work provides a proof-of-concept for a novel approach to using microwave signals to detect hydration states using sweat. The microwave-based sensor is composed of an ultra-light weight conformal antenna printed on a paper substrate and operating in the range 2–4 GHz. The proposed sensor structure can be placed directly on the skin and has an overall height of 230 μm. The performance of the antenna changes when liquids are absorbed by the substrate, and we demonstrate that these changes are linked to the sodium chloride concentrations and amount of solution absorbed. This is realized by measuring location and magnitude of the resonant frequency of the reflected microwave signal. After presenting the antenna design in Section 2.1, the approach to sensing is discussed in Section 2.2. Experimental results and case studies, including NaCl solutions and artificial sweat, are presented in Section 3, and concluding discussions are provided in Section 4.

## 2. Materials and Methods

### 2.1. Sensor Design

**Figure 1:**
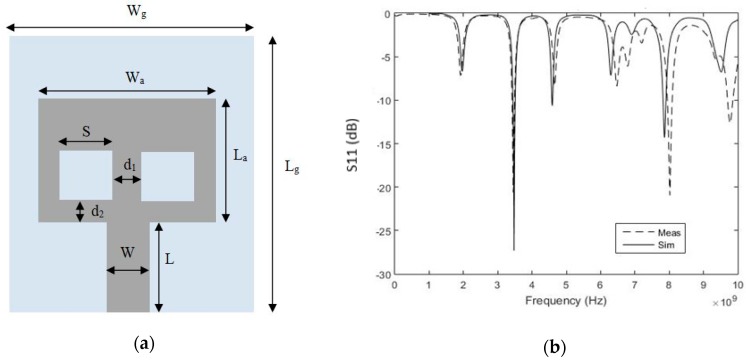
(**a**) Front View of paper-based antenna. (Ground: *W_g_* = 50 mm, *L_g_* = 60 mm, Antenna: *W_a_* =38 mm, *L_a_* = 29.5 mm, Feed Line: *W* = 11 mm, *L* = 19 mm, Slots: *S* = 11 mm, *d_1_* = 8 mm, *d_2_* = 5 mm); (**b**) The proposed antenna S_11_ (dB) versus frequency (Dotted: Measured, Solid: Simulated).

In this work, a low cost, disposable and conformal sensor is presented. The sensor is a paper-based 60 mm × 50 mm patch antenna as shown in Figure 1a. Various conformal, flexible antennas [37] have been designed on plastic (Polyethylene terephthalate (PET)and Kapton polyimide [38,39,40]), textiles and fluidic metal, as well as paper substrates [41]. Though plastic substrates have ultra-low height (40–50 μm), they are neither biodegradable nor recyclable. In addition, plastic substrates have higher cost compared to paper ones. Textile and fluidic metal-based substrates involve fabrication complexities [37]. The paper substrate is a good alternative in terms of cost, losses and fabrication complexity compared to existing flexible substrates. In addition, paper has the ability to absorb liquids and thus can be used for easy sampling of solutions such as sweat [22,42].

The antenna-based sensor is implemented by placing the paper substrate between the copper ground plane and copper patch antenna. The utilized substrate is cellulose filter paper (Whatman Grade 1 Qualitative Cellulose Filter paper, GE Healthcare Life Sciences, Mississauga, ON, Canada) of height 180 μm. The patch and ground plane are fabricated using non-conductive adhesive copper foil. The thickness of the copper tape is 40 μm and the adhesive thickness is 26 μm. The dielectric properties of the filter paper are measured using an 87050E Dielectric Probe (Agilent, Santa Clara, CA, USA) in contact with a stack of filter paper. To account for the adhesive layer, dielectric properties of the substrate are varied in simulation with initial values of *ε_r_* =1.4 and loss (tan δ) = 0.01 (recorded from dielectric probe measurements for solely filter paper). The dielectric properties of filter paper along with adhesive layer are calculated as 1.9 with loss (tan δ) of 0.025 by comparing simulated reflection coefficient to measurement of the fabricated prototypes. The estimated loss for the given cellulose filter paper substrate is almost three times lower than reported traditional paper substrates with tan δ of 0.06–0.07 [37,41]. Moreover the fabrication process for the proposed paper-based antenna does not involve paper preparation, heating treatment, lithography, etching, or UV exposure [41,43,44,45].

The patch antenna is designed with dimensions of 29.5 mm × 38 mm for a resonance of 3.5 GHz. The antenna has two square slots of length 11 mm as shown in Figure 1a. The two slots are incorporated to help absorb saline solutions or sweat. Absorbed solutions with different characteristics alter the substrate dielectric properties and thus reflection measurements will change accordingly. The antenna is fed by an 11 mm × 19 mm transmission line.

The paper antenna is modeled and optimized in the range of 1–5 GHz using simulation tools (CST Microwave Studio and Sim4life). The antenna shows a resonance of 3.48 GHz with simulated reflection coefficient of −27.3 dB. The patch antenna is fabricated and measured using a network analyzer (Agilent E8364B, Santa Clara, CA, USA). Measurements are compared to simulation results in Figure 1b. The antenna has measured reflection coefficient of −20.6 dB at 3.45 GHz. The antenna has measured bandwidth of 60 MHz compared to 70 MHz from simulation.

**Figure 2:**
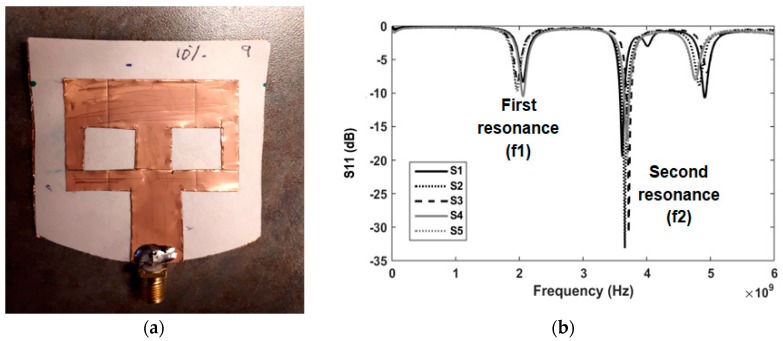
(**a**) Front View of fabricated sensor; (**b**) Measured S_11_ (dB) versus frequency for five sensors.

For sensor testing and validation, more than 40 sensors (same as shown in Figure 2a) were fabricated. The reflection coefficient (S_11_) for five sensors is shown in Figure 2b as an example to confirm consistency of sensor output. Although manufactured by hand using a regular cutter and scissors, the five sensors show a maximum variation of 100 MHz for the first resonance around 2 GHz and the second resonance around 3.5 GHz. Although 2 GHz is not the dominant resonance, data are recorded in this band due to significant variations noted after applying test solutions.

### 2.2. Sensing Methodology and Test Solutions

The key idea in this work is to use the paper substrate to absorb moisture, which then alters the dielectric constant of the paper. To test this concept, saline and artificial sweat solutions are applied to the sensor’s square slots in different quantities. The frequency of minimum reflection and corresponding magnitude of the reflection coefficient (S_11_) in decibels (dB) are recorded and compared before and after applying different solutions. The differences between these two recorded measurements for dry and wet sensors are related to changes in substrate dielectric properties. After sweat absorption, the sensor is taken off-body for post-absorption measurement. Therefore, the sensor behavior is not influenced by contact with the skin or tissues. This approach may be used clinically to collect a sample during an exam, so both the amount of sweat and content are of interest.

For the purpose of hydration monitoring using sweat, the proposed sensor uses microwave signals to track variations or different concentrations of one of the major components of sweat electrolytes, sodium chloride (NaCl). For both real [46] and artificial sweat [19,47,48,49,50], NaCl represents almost 65% of sweat electrolytes over a range of different hydration states [15,19]. 

Similar to previously proposed sensors in [9,17,19,20,21,22,33], initial testing and calibration incorporate application of NaCl solutions with known concentrations. The tested solutions reflect the NaCl concentrations described in the artificial sweat standard in [19,47,48,49,50], specifically concentrations from 8.5 to 200 mmol/L and extended to extreme cases of zero NaCl (representing hyponatremia) and 1.7 mol/L (representing severe hypernatermia and beyond). The solutions are composed of a base of 250 g of distilled water and mimic severe hypernatremia [9,10,11] (10% solution using 25 g (1.7 mol/L) NaCl), moderate hypernatremia [9] (0.5–2% solution using 1.25–6 g (85–410 mmol/L) NaCl)) and hyponatremia [9,12] using pure distilled water. 

The second phase of testing incorporates solutions mimicking sweat collected from hydrated (termed diluted sweat) and dehydrated individuals. According to the European standard (EN1811:2011) [19,49,50], artificial sweat for dehydrated individuals can be synthesized by dissolving 85 mmol of NaCl, 13 mmol of KCl, 17 mmol of lactic acid and 16 mmol of urea in one liter of deionised or distilled water. The weight ratios are 0.5% of NaCl (5 g), 0.1% of KCl (1 g), 0.1% of urea (1 g) and 0.1% of lactic acid (1.5 mL). Other recipes [47] adopt a higher weight ratio of 1% for NaCl (11 g/0.2 mol/L) to represent dehydration status. For diluted artificial sweat, the proportion of components is kept in the same ratios but with one tenth of the given dehydration concentrations. Normal or diluted sweat is composed of 0.5 g (0.05%) of NaCl, 0.1 g (0.01%) of KCl, 0.1 g (0.01%) of urea and 0.15 mL of lactic acid (0.01%). 

## 3. Results

Sensor performance is tested with different amounts and concentrations of saline solutions in Section 3.1, Section 3.2 and Section 3.3. In Section 3.4, the sensor is tested with solutions representing diluted and dehydrated sweat. Specifically, the ability of the sensor to detect changes in concentrations of sodium chloride and other electrolytes is explored, along with the sensor response to different amounts of samples representing different hydration states.

### 3.1. Detection of Applied Solution Quantity

**Figure 3:**
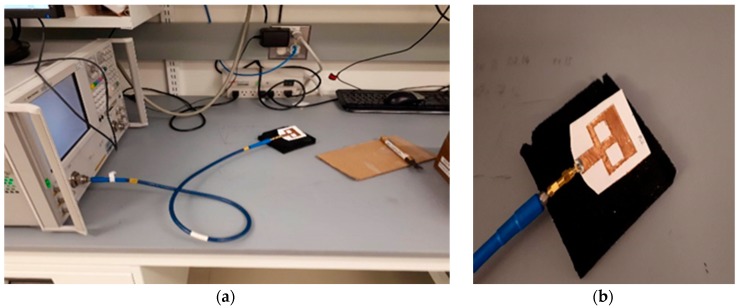
(**a**) Measurement set up (composed of Network Analyzer connected to the antenna-based sensor through SMA cable); (**b**) Detailed view for antenna-based sensor during measurements.

**Figure 4:**
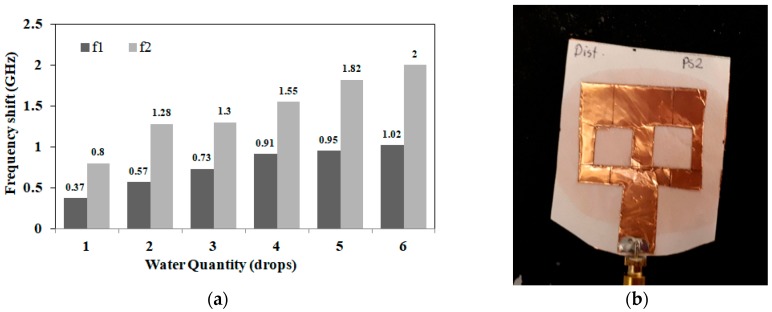
(**a**) Measured frequency shift for both bands (f1: first resonance, f2: second resonance) versus applied water quantities at 2% NaCl (1 drop = 0.05 mL); (**b**) Sensor photo after absorbing 6 drops (0.3 mL) of saline solution.

In this section, the proposed sensor is tested in terms of detecting the amount of solution. Six new sensors (similar to those shown in Figure 2) were prepared and different amounts of 2% NaCl solution (0.4 mol/L) were applied to each sensor. Quantities ranging from 1 drop to 6 drops (equivalent to 0.05 mL to 0.3 mL) were tested using the measurement set up shown in Figure 3. Different frequency shifts were recorded for different quantities as shown in Figure 4a. By calibrating the results, it could be deduced that an average shift in frequency of 0.14 GHz per drop (2.8 MHz/μL) is recorded for the first band and 0.3 per drop (6 MHz/μL) for the second band. The solution quantity test was repeated blind, where both the frequency shift and amount of applied water were set and collected separately. By analyzing the induced shift, the amount of applied water was successfully detected for all sensors.

### 3.2. Detection of Salt Concentrations

**Figure 5:**
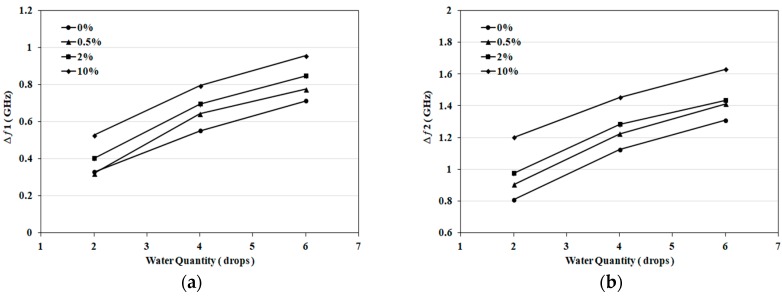
(**a**) Frequency shift for the first band at 2 GHz versus applied water quantity at different salt concentrations (1 drop = 0.05 mL); (**b**) Frequency shift for the second band at 3.5 GHz versus applied water quantity at different salt concentrations (1 drop = 0.05 mL).

The following set of experiments incorporates NaCl solutions with concentrations of 0%, 0.5%, 2% and 10% representing different hydration states. To confirm consistency of results, 5 sets of sensors were prepared. Each set is composed of four newly fabricated sensors. Each sensor is tested with a different salt concentration and at different quantities. Saline solution is applied in 3 consecutive rounds of 2 drops (0.1 mL) each. Reflection coefficient magnitude (S_11_) in dB is recorded before and after each round of application of saline. In this experiment, a total of 6 drops (0.3 mL) is applied to a single sensor. Figure 4b shows a photo for the proposed sensor after absorbing 6 drops (0.3 mL) of saline solution and covering 80% of substrate area. The frequency shifts at different salt concentrations and quantities are shown in Figure 5a,b. Figure 5a presents results for the first resonance around 2 GHz, while Figure 5b presents results for the second band (main resonance) around 3.5 GHz.

Results shown in Figure 5 represent the average of five repetitions of the whole set of experiments. The consistency of results is verified through five rounds of measurements with maximum variation of ±0.068 GHz at 0%, ±0.1 GHz at 0.5%, ±0.072 GHz at 2%, and 0.096 GHz at 10% at the first resonance. For the second resonance, maximum measurement variations of ±0.14 GHz at 0%, ±0.16 GHz at 0.5%, ±0.18 GHz at 2%, and ±0.1 GHz at 10% are recorded.

**Figure 6:**
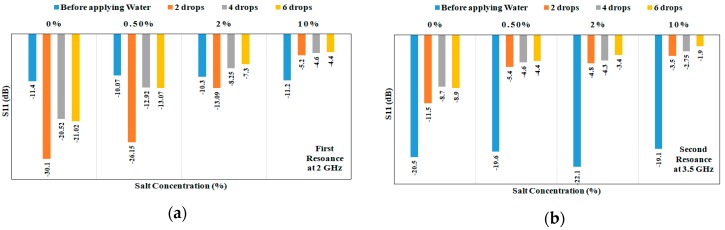
(**a**) Measured S_11_ in dB at different solution quantities (1 drop = 0.05 mL) and salt concentrations for the first resonance around 2 GHz; (**b**) Measured S_11_ in dB at different quantities (1 drop = 0.05 mL) and salt concentrations for the second resonance around 3.5 GHz.

From the results in Figure 5, the frequency shift increases with increasing saline quantity at all concentrations. It can be also deduced that each salt concentration has a frequency shift characteristic that typically does not intersect with other concentrations. Finally, frequency shifts are higher in Figure 4a compared to Figure 5. This can be explained as results recorded in Figure 5a,b involve water evaporation. To gain further insight into sensitivity, the magnitude of S_11_ was recorded for different solution quantities and at different salt concentrations, as shown in Figure 6a,b. Higher S_11_ magnitudes are measured with increasing quantities and salt concentrations. For the first band at 2 GHz, a magnitude span of 18 dB with changing salt concentrations from 0% to 10% (0–1.7 mol/L) is presented in Figure 6a. For the second band at 3.5 GHz, a magnitude span of 8 dB is recorded in Figure 6b. 

### 3.3. Case Studies: NaCl Solutions

The trends noted in Figure 5a,b suggest detecting salt concentration in the range 0–1.7 mol/L and applied solution quantity using the frequency shift at both bands. Ten case studies that test this concept are explained in detail. First, the detection range is divided into three ranges of NaCl concentrations. These ranges are 0–0.5%, 0.5–2% and greater than 2% (2–10%). The given ranges reflect different hydration states. Second, for each given sensor, the magnitude of S_11_ in dB is recorded before and after applying a predefined quantity and concentration of salt solution. The shift in frequency is recorded for the first band at 2 GHz and the second band at 3.5 GHz.

**Figure 7:**
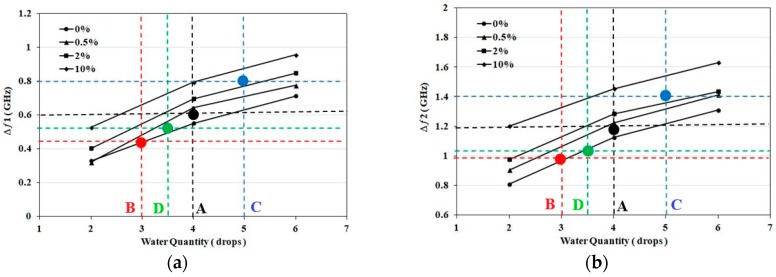
(**a**) Detected Frequency shift for the first band at 2 GHz for NaCl Samples: A (Black), B (Red), C (Blue), D (Green); (**b**) Detected Frequency shift for the second band at 3.5 GHz for NaCl Samples: A (Black), B (Red), C (Blue), D (Green).

From the results shown in previous sections, an average frequency shift of *Δ_1_* = 2.8 MHz/μL is recorded for the first band and *Δ_2_* = 6 MHz/μL for the second band. Thus by using the measured frequency shifts and average frequency shift per drop or mL, the amount of applied solution is estimated. By using Figure 5a,b with the estimated quantity, the salt concentration range is estimated. The sensing procedure for one of the samples (Sample A) is described as follows: Sample A represents application of four drops (0.2 mL) of 0.5% NaCl (0.1 mol/L) solution to a sensor. The recorded shift for the first band is *Δƒ_1_* = 0.58 GHz and for second band is Δƒ_2_ = 1.17 GHz. Using *Δf_1_*, the corresponding amount of NaCl solution calculated from the first band is *N_1_* = *Δƒ_1_*/*Δ_1_* = 0.58/2.8 = 0.21 mL (4.1 drops). For the second band, the corresponding amount is *N_2_* = *Δƒ_2_*/*Δ_2_* = 1.17/6 = 0.195 mL (3.9 drops). From *N_1_* and *N_2_*, an average of 0.2 mL (*N_avg_* = 4 drops) of salt water is calculated as the applied quantity for the given sensor. Using frequency shift values and four drops solution quantity, a salt concentration in the range 0–0.5% (closer to 0.5% line) is estimated using Figure 5. The given concentration result is confirmed from both charts of the two recorded bands. Numerical data for 10 saline samples applied to 10 new sensors with different quantities and concentrations are summarized in Table 1. Samples A–D are shown in Figure 7a,b as exemplary sensing cases using detecting charts.

### 3.4. Artificial Sweat Trials

The work presented in the previous section validates sensor operation to differentiate between NaCl solutions with concentrations in the range 0–10% (0–1.7 mol). In the following set of experiments, artificial sweat with different electrolyte concentrations, representing diluted and dehydrated sweat, is applied to the proposed sensor. 

**Figure 8:**
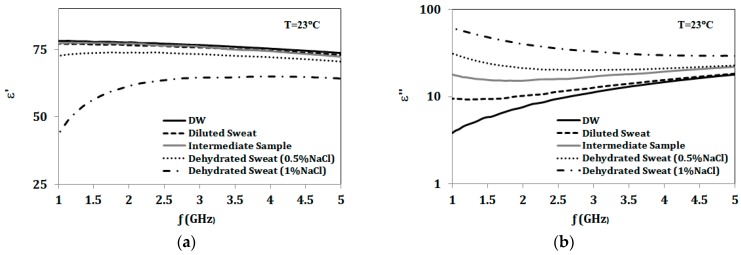
(**a**) Dielectric constant (ε’); (**b**) Dielectric loss (ε″) for distilled water (DW), diluted sweat (0.05% NaCl, 0.01% for each urea, lactic acid and KCl), intermediate sample (0.05% NaCl, 0.1% for each urea, lactic acid and KCl), and dehydrated sweat (0.5% & 1% NaCl, 0.1% for each urea, lactic acid and KCl).

Significant changes in dielectric properties of sweat are expected with varying concentrations of the dominant electrolyte, NaCl, compared to other electrolytes [51,52,53]. For validation, dielectric properties for both diluted and dehydrated artificial sweat were measured using an 87050E Dielectric Probe (Agilent Santa Clara, CA, USA). Dielectric constant (ε’) and losses (ε″) are recorded and compared for artificial sweat and distilled water in the sensor band of 1–5 GHz. From the measurements shown in Figure 8, diluted sweat with 0.01 mol of NaCl almost has same dielectric constant as distilled water over the sensor band. However, diluted sweat exhibits greater loss in the lower frequencies of the band, specifically we note higher loss at 2 GHz compared to 3.5 GHz. Dielectric properties are recorded as well for NaCl concentrations of 0.1 mol/L and 0.2 mol/L, representing concentrations for dehydration. The measured properties show greater differences at 2 GHz compared to 3.5 GHz. Thus, sensor sensitivity is expected to be higher at the first resonance compared to the second one. Moreover, the measured dielectric properties of prepared artificial sweat mixtures based on the European standard shows similarity in values with those recorded for real sweat in [54].

**Figure 9:**
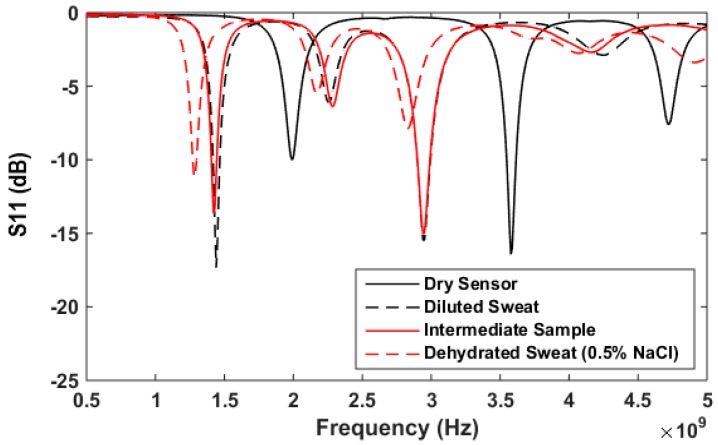
S_11_ in dB versus frequency for proposed sensor and different solutions with fixed volume of 200uL. Solutions applied to sensors: diluted sweat (0.05% NaCl, 0.01% for each urea, lactic acid and KCl), intermediate sample (0.05% NaCl, 0.1% for each urea, lactic acid and KCl), and dehydrated sweat (0.5% NaCl, 0.1% for each urea, lactic acid and KCl).

Next, the sensor response after application of artificial sweat mixtures with a range of NaCl concentrations is explored. This includes using diluted sweat (0.05% NaCl, 0.01% for each urea, lactic acid and KCl) and dehydrated sweat (0.5% NaCl, 0.1% for each urea, lactic acid and KCl) as test solutions. To validate the sensitivity of the proposed sensor to NaCl in the presence of other sweat components, an additional sample is synthesized with the same NaCl concentration (0.05%) as diluted sweat, while other sweat electrolytes concentrations are increased to mimic dehydrated sweat concentrations (0.1% for each urea, lactic acid and KCl). Dielectric properties for this sample (termed intermediate) are also recorded and shown in Figure 8. Three new sensors are prepared and tested with fixed volumes of 200 μL of diluted sweat (0.05% NaCl), the intermediate sample and dehydrated sweat (0.5% NaCl). Figure 9 compares the S_11_ response for sensors with different tested solutions. The given results confirm the frequency shift with changing NaCl concentration. In addition, the S_11_ response shows the same frequency shift when keeping the NaCl concentration same as diluted sweat while increasing other sweat electrolytes. This suggests that the sensor’s frequency response is dominated by the NaCl concentration.

Next, we further explore the ability of the proposed sensor to differentiate between different amounts of sweat representing diluted and dehydrated states. Three solutions are tested, representing diluted sweat, dehydrated sweat with 0.5% NaCl and dehydrated sweat with 1% NaCl. Each of the three solutions is applied to a sensor in increments of two drops, and the changes in resonant frequency and magnitude of S_11_ are noted after application of two, four and six drops. This test is repeated using a total of five sensors for each solution, and the results are averaged.

**Figure 10:**
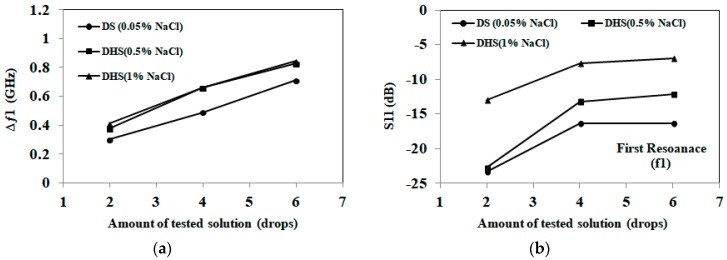
(**a**) Detected frequency shift for the first band at 2 GHz for artificial sweat samples; (**b**) Detected S_11_ in dB for the first band at 2 GHz for artificial sweat samples (1 drop =0.05 mL). Diluted sweat (DS) has a NaCl concentration of 0.05% (0.01 mol/L), while dehydrated sweat (DHS) has a NaCl concentration in the 0.5–1% (0.1–0.2 mol/L) range.

**Figure 11:**
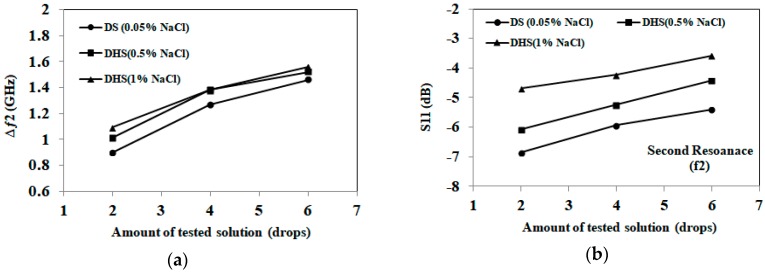
(**a**) Detected frequency shift for the second band at 3.5 GHz for artificial sweat samples; (**b**) Detected S_11_ in dB for the second band at 3.5 GHz for artificial sweat samples (1 drop = 0.05 mL). Diluted sweat (DS) has a NaCl concentration of 0.05% (0.01 mol/L), while dehydrated sweat (DHS) has a NaCl concentration in the 0.5–1% (0.1–0.2 mol/L) range.

The results for the different amounts of absorbed artificial sweat are shown in Figure 10 and Figure 11. The consistency of these results is verified through five rounds of measurements with maximum variation of ±0.08 GHz for diluted sweat, ±0.1 GHz for dehydrated sweat with 0.5% NaCl, ±0.046 GHz for dehydrated sweat with 1% NaCl at the first resonance. For the second resonance, maximum measurement variations of ±0.08 GHz for diluted sweat, ±0.092 GHz for dehydrated sweat with 0.5% NaCl, ± 0.046 GHz for dehydrated sweat with 1% NaCl are recorded. 

From the results, we note that distinct frequency shifts are recorded for diluted (0.05% NaCl) and dehydrated sweat (0.5–1% NaCl) at both bands. However, frequency shift alone is not capable of distinguishing between dehydrated sweat with 0.5% NaCl and 1% NaCl concentrations. On the other hand, the magnitude of S_11_ shows distinct levels for each NaCl concentration of diluted and dehydrated sweat at both bands. Greater changes in the detected S_11_ magnitude are recorded at the first band at 2 GHz compared to second band at 3.5 GHz. These changes are aligned with measurements of dielectric properties of tested solutions. 

By comparing artificial sweat results with saline solutions, sensors retain similar trends and frequency shifts for the same NaCl concentrations and amounts of tested solutions. On the other hand, S_11_ magnitude values slightly change (±0.6 dB at both frequencies) with the presence of other electrolytes in the artificial sweat mixtures. Moreover, the trends noted in Figure 10 and Figure 11 could be used to develop hydration sensing charts, as well as machine learning approaches for the proposed paper-based antenna. These approaches would detect NaCl concentration and applied solution quantity using the frequency shift and S_11_ magnitude at both bands.

## 4. Discussion

This paper presents a demonstration of how microwave signals can be used to distinguish between diluted and dehydrated sweat. The conformal, low cost, disposable paper-based sensor uses reflection-based measurements to detect different hydration states using sweat. The sensing approach is based on the absorption of sweat/NaCl solution by the cellulose filter paper substrate. Different hydration states may result in sweat with different dielectric properties and conductivities due to changes in concentration of sodium chloride (and other electrolytes). These differences in sodium concentration are detected as different frequency shifts and magnitude levels in reflection coefficient measurements. Thus, different hydration states may be detected. Moreover, the sensor succeeds in detecting the amount of solution applied. 

Several experiments have been conducted with the given sensor and solutions at different NaCl concentrations (0–10% or 0–1.7 mol/L) and quantities to validate sensor operation. Through the measurements, detected NaCl concentration ranges are classified as 0–0.5%, 0.5–2% and >2%. These ranges represent hyponatremia, moderate hypernatremia and severe hypernatremia hydration status. Microwave measurements show the success of the proposed sensor to distinguish between different concentrations of sodium chloride in the range from 0 to 1.7 mol/L (0–10%) with RL magnitude span of 8–18 dB and ƒ_min_ changes of 430 MHz. The results show maximum variation of ±0.1 GHz at the first resonance and ±0.18 GHz at the second resonance for all concentrations. Moreover, the amount of solution is detected with accuracy of ±0.05 mL. 

The proposed sensor was also tested with artificial sweat at different electrolyte concentrations and at different frequency bands. Microwave measurements have demonstrated the capability of the sensor to differentiate between diluted and dehydrated sweat with sodium chloride concentrations in the range from 0.01–0.2 mol/L (0.05–1%). The corresponding changes in RL magnitude span 2–10 dB and ƒ_min_ changes span 170 MHz. The results show maximum variation of ±0.08 GHz for diluted sweat and ±0.1 GHz for dehydrated sweat. Moreover, the amount of absorbed artificial sweat is also detected with accuracy of ±0.05 mL. More significant changes in dielectric properties of artificial sweat are noted and measured with varying NaCl concentrations in comparison to property changes obtained when varying concentrations of other major sweat electrolytes (e.g. potassium chloride (KCl), urea and lactic acid). Therefore, the most significant dielectric property changes in sweat are likely to be related to changes in NaCl.

Compared to previously reported microwave-based hydration sensors [9,34,35,36], the proposed sensor demonstrates for the first time the capability of microwave signals to track sweat electrolytes for hydration monitoring. The proposed work compares the response and sensitivity of the proposed sensor as well as measurement variations at different operating frequencies (2 and 3.5 GHz) and at different tested solutions. The given sensor shows almost four times higher sensitivity (10 dB at different artificial sweat concentrations or 18 dB at different concentrations of NaCl solutions) than other reported microwave hydration sensors (3.4 dB at different concentrations of NaCl solutions [9]). Such enhanced sensitivity is expected to provide detection accuracy and sensitivity when testing patients with low sweat secretion.

The proposed sensor has an overall size of 50 mm × 60 mm, which is appropriate for sampling sweat at different locations (e.g. armpit, forehead, forearm or chest). Operating at 3.5 GHz allows designing the sensor with adequate slot size incorporated for collecting sweat/salt solution. The cellulose filter paper substrate is highly conformal with overall height of 230 μm, low loss and environmentally friendly compared to existing flexible substrates. Microwave measurements show that, for the given sensor, no changes occur when absorbing more than 0.4–0.5 mL (8–10 drops), corresponding to covering the whole substrate with solution. The given sensor does not require conditioning time and provides instantaneous response. Detection time per measurement, including processing 2000 points, was less than 2 min. With the given substrate, using copper foil and no fabrication complexities, the overall sensor cost is estimated at 15–20 cents. This price point can place the proposed sensor in the disposable category. 

## 5. Conclusion

This paper demonstrates for the first time the capability of microwave signals to track sweat electrolytes for hydration monitoring. The conformal, low cost, disposable, non-wearable, paper-based sensor uses reflection-based measurements to differentiate between diluted and dehydrated sweat. It also demonstrates high sensitivity to NaCl in the presence of other major sweat constituents: potassium chloride (KCl), urea and lactic acid. Thus, the NaCl concentration level in sweat mixtures could be also estimated. The given sensor shows almost 4 times higher sensitivity compared to other reported microwave hydration sensors. Such enhanced sensitivity is expected to provide detection accuracy and sensitivity when testing patients with low sweat secretion.

The proposed design can be directly placed on skin in the current form, absorbing sweat, without changing measurement procedures. Thus, the sensing decision is not influenced by contact with the skin or tissues and does not require proximity to the human body. The proposed sensor has the advantages of consistent performance, high sensitivity, simple sweat sampling, no fabrication complexities, as well as low price point which is appealing for integration in clinical practices. 

## Figures and Tables

**Table 1:** Summary of samples A–J.

Samples	First Band (GHZ)	Second Band (GHZ)	Detection Decision
Sample. A (4 drops, 0.5%)	Δƒ_1_=0.58	Δƒ_2_=1.14	N_avg_=4 (0–0.5%)
Sample. B (2 drops, 0%)	Δƒ_1_=0.44	Δƒ_2_=0.97	N_avg_=3 (0–0.5%)
Sample. C (4 drops, 10%)	Δƒ_1_=0.8	Δƒ_2_=1.4	N_avg_=5 (2–10%)
Sample. D (3 drops, 0%)	Δƒ_1_=0.51	Δƒ_2_=1.03	N_avg_=3.5 (0–0.5%)
Sample. E (6 drops, 2%)	Δƒ_1_=0.92	Δƒ_2_=1.43	N_avg_=7 (2–10%)
Sample. F (5 drops, 2%)	Δƒ_1_=0.8	Δƒ_2_=1.39	N_avg_= 5 (2–10%)
Sample. G (6 drops, 0%)	Δƒ_1_=0.72	Δƒ_2_=1.33	N_avg_=6 (0–0.5%)
Sample. H (3 drops, 0.5%)	Δƒ_1_=0.47	Δƒ_2_=1.14	N_avg_=3.5 (0–0.5%)
Sample. I (5 drops, 10%)	Δƒ_1_=0.9	Δƒ_2_=1.51	N_avg_=6 (2–10%)
Sample. J (4 drops, 2%)	Δƒ_1_=0.62	Δƒ_2_=1.2	N_avg_=4 (0.5–2%)
